# Patients’ Experiences of Using Skin Self-monitoring Apps With People at Higher Risk of Melanoma: Qualitative Study

**DOI:** 10.2196/22583

**Published:** 2021-08-13

**Authors:** Emily Habgood, Christopher McCormack, Fiona M Walter, Jon D Emery

**Affiliations:** 1 Department of General Practice and Centre for Cancer Research University of Melbourne Melbourne Australia; 2 Department of Surgical Oncology Peter MacCallum Cancer Centre Melbourne Australia; 3 Primary Care Unit, Department of Public Health and Primary Care University of Cambridge Cambridge United Kingdom; 4 Wolfson Institute of Population Health Queen Mary University of London London United Kingdom

**Keywords:** melanoma, skin cancer, early detection, mobile apps, qualitative, mHealth, mobile phone

## Abstract

**Background:**

Melanoma is the fourth most commonly diagnosed cancer in Australia. Up to 75% of melanomas are first detected by patients or their family or friends. Many mobile apps for melanoma exist, including apps to encourage skin self-monitoring to improve the likelihood of early detection. Previous research in this area has focused on their development, diagnostic accuracy, or validation. Little is known about patients’ views and experiences of using these apps.

**Objective:**

This study aims to understand patients’ views and experiences of using commercially available melanoma skin self-monitoring mobile apps for a period of 3 months.

**Methods:**

This qualitative study was conducted in two populations: primary care (where the MelatoolsQ tool was used to identify patients who were at increased risk of melanoma) and secondary care (where patients had a previous diagnosis of melanoma, stages T0-T3a). Participants downloaded 2 of the 4 mobile apps for skin self-monitoring (SkinVision, UMSkinCheck, Mole Monitor, or MySkinPal) and were encouraged to use them for 3 months. After 3 months, a semistructured interview was conducted with participants to discuss their experiences of using the skin self-monitoring mobile apps.

**Results:**

A total of 54 participants were recruited in the study, with 37% (20) of participants from primary care and 62% (34) from secondary care. Interviews were conducted with 34 participants when data saturation was reached. Most participants did not use the apps at all (n=12) or tried them once but did not continue (n=14). Only 8 participants used the apps to assist with skin self-monitoring for the entire duration of the study. Patients discussed the apps in the context of the importance of early detection and their current skin self-monitoring behaviors. A range of features of *perceived quality* of each app affected engagement to support skin self-monitoring. Participants described their skin self-monitoring routines and potential mismatches with the app reminders. They also described the technical and practical difficulties experienced when using the apps for skin self-monitoring. The app’s positioning within existing relationships with health care providers was crucial to understand the use of the apps.

**Conclusions:**

This study of patients at increased risk of melanoma highlights several barriers to engagement with apps to support skin self-monitoring. The results highlight the wide-ranging and dynamic influences on engagement with mobile apps, which extend beyond app design and relate to broader contextual factors about skin self-monitoring routines and relationships with health care providers.

## Introduction

Skin cancers account for 80% of all newly diagnosed cancers in Australia, with melanoma being the most harmful [1]. Early detection is key, as it provides a better chance of receiving timely treatment. More than 91% of Australians survive if their melanomas are detected early [1]. Up to 75% of melanomas are first detected by patients or their family or friends [2]. Encouraging people to self-monitor their skin for suspicious moles on their bodies could encourage early diagnosis [2]. Current guidelines already recommend Australians at increased risk of melanoma to monitor their own skin in between appointments [3,4]. However, there is currently little information on how patients are recommended to do this, and many patients are completing this insufficiently [5].

Mobile apps for melanomas are becoming increasingly popular. There is an abundance of commercially available mobile apps for melanoma across the different mobile app stores [6,7]. The purpose of these apps varies from prevention (UV exposure apps) to treatment management (drug and side effect management). Apps that encourage skin self-monitoring are designed to support the early detection of melanoma by identifying changes in moles. Most research into these mobile apps for skin self-monitoring for melanoma has focused on their development or diagnostic accuracy [8-11], but there have been limited studies on the actual use of these apps outside controlled *laboratory* settings. Qualitative research provides a deeper understanding of people’s experiences, thoughts, and opinions to explore what determines the effective implementation of digital interventions [12,13]. Recent research has shed light on patients’ perceptions of the use of mobile health apps for melanoma. Specifically, Koh et al [14] found that patients had positive views about apps for skin self-monitoring and thought they would benefit from using them, but this was based on the intended use of a hypothetical app. We suggest that allowing participants to experience using these types of apps over a period of time provides greater ecological validity. In this study, we aim to understand users’ experiences and use of skin self-monitoring mobile apps for melanoma over a 3-month period, focusing on people who were at increased risk of melanoma, as this is consistent with the current Australian guidelines [3,4].

More specifically, we aim to understand participants’ experiences of actually using these mobile apps and the reasons they chose whether to use and engage with the apps. We also wanted to determine if this was a potentially feasible way to recruit people at risk of melanoma in future studies of skin self-monitoring apps.

## Methods

### Study Design and Ethics Approval

This study used a qualitative design with a 3-month follow-up period using a baseline questionnaire and semistructured interviews. A 3-month period was considered to be sufficient to understand participants’ interest and patterns of use of the mobile apps and understand their experiences. Participants downloaded the apps on their own phones, were provided with a brief demonstration of both apps, and received automated reminders once per month via each app. Semistructured interviews were used to understand participants’ thoughts and experiences after the 3-month period of ad libitum use of 2 of the 4 apps allocated to them on the basis of their phone’s operating system (iOS or Android). This study was reviewed and approved by the University of Melbourne Health Sciences Human Ethics Committee (1749081) and the Peter MacCallum Cancer Centre Human Research Ethics Committee (HREC/17/PMCC/214).

### Recruitment

#### Study Setting

Participants were recruited from two different populations: Melbourne general practices, where participants were identified as at increased risk of melanoma on the basis of risk factors; and the Melanoma Outpatient Clinic at Peter MacCallum Cancer Centre (Melbourne, Australia), where participants had a previous diagnosis of melanoma. The study was conducted between October 2018 and February 2019.

#### Primary Care

In primary care, recruitment was undertaken across 3 busy Melbourne general practices. All patients in the waiting room of the practices were consecutively approached and invited to complete the MelatoolsQ tool [15] to determine if they were eligible. Patients were excluded if they were aged <18 years, unable to understand English, or acutely unwell.

The MelatoolsQ tool is a self-completed survey, which is delivered on an iPad. It contains a modified version of the Williams melanoma risk prediction model [16], which includes the following risk factors: sex, age, natural hair color at the age of 15 years, number of raised moles on both arms, the density of freckles on both arms before the age of 20 years, number of severe sunburns up to the age of 18 years, and previous nonmelanoma skin cancer (basal cell carcinoma and squamous cell carcinoma). A melanoma risk score was calculated from the patients’ responses; if they scored 25 or more, they were categorized as increased risk of melanoma and invited to participate in the study [15,16].

#### Secondary Care

In secondary care, all patients attending an outpatient appointment for their current or previous early-stage melanoma (stages T0-T3a) and aged 18 years or older were approached and invited to participate in the study. Patients were ineligible if they had suspicion or evidence of metastatic disease or were receiving palliative treatment.

### Procedure

All participants who were eligible from either primary or secondary care were invited to participate. The aims of the study were discussed, and all participants were provided with a plain English statement explaining the details of participation. Participants recruited to the study had to own a compatible smartphone (Android or iOS operating system) and have sufficient data storage on their phone to download and store photographs (approximately 130 MB). Written consent was obtained from all participants before completing a short baseline survey.

Consented participants were assisted in downloading 2 study apps onto their own phones, which were dependent on their phone’s operating system. They were provided with a short booklet and demonstration of how to use each app. Participants were asked to use the apps at least once a month for the 3 months of the study, with a monthly SMS text reminder to check their moles through the app.

### Data Collection

A baseline questionnaire collected data on demographics and patterns of mobile phone use. All participants were invited to participate in a telephonic semistructured interview at the end of the 3-month time point, which was audio recorded. The interview guide was designed to explore participants’ experiences and preferences for using the apps and their skin monitoring behaviors (Multimedia Appendix 1).

### App Selection

The melanoma skin self-monitoring mobile apps identified for the study were SkinVision, UMSkinCheck, Mole Monitor, and MySkinPal. The researchers have no association with the development or marketing of these apps. Inclusion criteria for app selection were apps that were designed for patient use, allowed users to take photographs of their skin within the app, compare photographs over time, and had built-in reminder notifications and information on skin self-monitoring.

The selected apps were identified through a previous review of available mobile apps designed for early detection of melanoma [7]. Kassianos et al [7] identified 39 apps available at that time on app stores for melanoma, and we selected 4 apps on the basis of their functionality. The apps varied by the operating system they were compatible with (either Android or iOS) and the level of assistance provided to determine changes between photos. The Mole Monitor and UMSkinCheck apps were only available on iOS at the time of the study. During the study period, there were no updates to 3 of the apps and minor bug fixes to SkinVision. The apps were allocated to participants depending on their phone’s operating system. We wanted to understand participants’ experiences of using a melanoma skin self-monitoring mobile app per se rather than the specific apps selected. Therefore, we decided to provide participants with 2 apps each (depending on their phone operating system) to allow comparison of app features and content but to minimize participant burden. We believed that this more closely reflected the usual consumer approaches to trialing new apps while studying those that had similar functionality to support skin self-monitoring.

### Data Analysis

Quantitative baseline data were collected using REDCap (Research Electronic Data Capture) [17] and analyzed using descriptive statistics with Stata Statistical Software (version 17, StataCorp LLC) [18].

Qualitative data were audio recorded and transcribed verbatim. Transcripts from the semistructured interviews were analyzed using inductive and deductive thematic analyses, using the stepped approach described by Braun and Clarke [19]. All coding was undertaken by EH, a health services researcher, with a subsample coded by JDE, an academic general practitioner (GP); discrepancies were discussed and resolved as a team. The team also included a second academic GP and a dermatologist. All individuals in the team brought their perspectives to the analysis. Data saturation was reached when the team agreed that no new themes were arising from the transcripts. All analyses were performed using Dedoose (version 8.3.17) [20].

## Results

### Participant Demographics

A total of 54 participants (28/54, 52% female; mean age 57.3 years, SD 12.5 years) were recruited in the study between June and September 2018. A total of 20 participants were recruited from primary care and 34 from secondary care. Among the 54 participants who completed the baseline questionnaire, 34 (63%) were interviewed about their experiences (12 from primary care and 22 from secondary care). The demographic characteristics are presented in Table 1. The median interview time was 21 (range 5-39) minutes. Nine participants were lost to follow-up, and 11 participants withdrew during the study period. The main reasons for withdrawal were competing health issues (n=3), difficulty using the apps (n=3), and being too busy to participate (n=2). These participants were mostly older and from rural areas.

Table 2 presents data on patterns of use of apps by participants. Of the 34 participants interviewed, 88% (30) had downloaded an app in the last year and 73% (25) often use the apps on their phone more than once a day. More than half of the participants (20/34, 59%) had health-related apps on their phone.

**Table 1:** Demographic characteristics (N=54).

Variable		Total recruited, n (%)	Total interviewed (n=34), n (%)	Total not interviewed (n=20), n (%)
**Gender**				
	Female	28 (52)	15 (44)	13 (65)
	Male	26 (48)	19 (56)	7 (35)
**Age (years)**				
	18-44	8 (15)	6 (18)	2 (10)
	45-54	9 (16)	6 (18)	3 (15)
	55-64	21 (39)	16 (47)	5 (25)
	65-74+	16 (30)	6 (17)	10 (50)
**Education**				
	Year 11 or below	15 (28)	8 (23)	7 (35)
	Year 12 or equivalent	8 (15)	5 (15)	3 (15)
	Trade or apprenticeship	10 (19)	7 (20)	3 (15)
	Tertiary certificate or diploma	6 (11)	4 (12)	2 (10)
	Undergraduate	4 (7)	2 (6)	2 (10)
	Postgraduate	11 (20)	8 (24)	3 (15)
**ARIA^a^ postcode classification**				
	City	36 (67)	30 (88)	6 (30)
	Rural	18 (33)	4 (12)	14 (70)
**Phone operating system**				
	iOS	36 (67)	21 (62)	15 (75)
	Android	18 (33)	13 (38)	5 (25)

^a^ARIA: Accessibility or Remoteness Index of Australia.

**Table 2:** Baseline survey responses (N=54).

Question		Total recruited, n (%)	Total interviewed (n=34), n (%)	Total not interviewed (n=20), n (%)
**Number of apps downloaded in the last year**				
	0	7 (13)	4 (12)	3 (15)
	1-4	16 (29)	9 (26)	7 (35)
	5-10	21 (39)	14 (41)	7 (35)
	11-20	8 (15)	6 (18)	2 (10)
	≥20	2 (4)	1 (3)	1 (5)
**What types of apps do you use on your phone?^a^**				
	Games	21 (39)	10 (29)	11 (55)
	Social networking	37 (69)	23 (68)	14 (70)
	Video or movies	2 (4)	2 (6)	0 (0)
	News	22 (41)	15 (44)	7 (35)
	Maps or navigation	45 (83)	28 (82)	7 (35)
	Weather	43 (80)	25 (73)	18 (90)
	Banking or finance	38 (70)	24 (71)	14 (70)
	Shopping or retail	19 (35)	11 (32)	8 (40)
	Health-related	20 (37)	13 (38)	7 (35)
**How often do you typically use the apps on your smartphone?**				
	More than 10 times a day	15 (28)	8 (23)	7 (35)
	2-10 times per day	24 (44)	17 (50)	7 (35)
	Once a day	8 (15)	4 (12)	4 (20)
	Less than once a day	7 (13)	5 (15)	2 (10)
**How many health-related apps do you have on your phone?**				
	0	24 (44)	14 (41)	10 (50)
	≥1	30 (56)	20 (59)	10 (50)

^a^More than 1 option could be chosen; on average, 4.5 were selected, with a median of 5.

### Use of the Skin Self-monitoring Apps

Overall, although a minority of the participants who were interviewed thought the skin self-monitoring apps were helpful and used them for the entire duration of the study (n=8), most participants either did not use the apps at all (n=12) or tried them once and did not continue (n=14). Participants spoke about their preferences for the different apps, which mostly referred to their user experience of the apps. Of the 4 apps used in the study, no app was preferred over the other by a majority of users. There were no discernible differences in views about the skin self-monitoring apps between those with a previous melanoma and those recruited from primary care. We present the results of the qualitative data analysis in relation to the following core themes: perceived benefits of early detection and experiences of skin self-monitoring, the experience of using the apps to support skin self-monitoring, skin self-monitoring routines and the role of app reminders, and the apps and their positioning within existing relationships with health care providers (HCPs). Data saturation was reached by the last 3 interviews, where no new themes were arising for both primary and secondary care participants. All relevant quotes are provided in Multimedia Appendix 2.

### Benefits of Early Detection

Many participants, regardless of their use, discussed the importance of early detection of melanoma and how these apps could support patients in identifying melanomas at an early stage. As a result, all participants who used the app throughout the study thought that using the app provided peace of mind and reduced some of the uncertainty about checking their skin for signs of melanoma. Despite recognizing the potential benefits of using apps for the early detection of melanoma, there were variable degrees of engagement with them. Some participants felt that they were more relevant to their needs, and this was driven, in part, by their perceived increased risk of melanoma.

### Experience of Skin Self-monitoring

In the context of the perceived benefits of early detection of melanoma, all participants discussed skin self-monitoring and recognized the importance of checking their own skin regularly. Some participants discussed the importance of routine to engage in regular skin self-monitoring, for example, performing it while they were dressing for the day. However, some participants felt that skin self-monitoring was not appropriate for them, describing the challenges of skin self-monitoring on having large numbers of moles and the challenges of deciding which ones to monitor, especially when they had many to choose from.

### Experience of Using Apps to Support Skin Self-monitoring

Although individual opinions on skin self-monitoring varied, most participants perceived skin self-monitoring positively and continued to perform it regardless of their engagement with the apps. Participants described several factors that influenced their perceptions of the quality of the apps, which affected their engagement with them. People were more likely to engage with an app that they felt was of high quality, although what exactly determined this perception differed among users. Primarily, users described the importance of intuitive design and the simplicity of use to foster engagement. This was key as the app was only recommended to be used once a month and not on a more frequent basis, as in most other apps.

When discussing their experience of the different apps, participants described the importance of simple navigation through the app and the ability to move through the app easily as they checked individual moles. Not surprisingly, key functions in the apps were considered better in some apps compared to others—a critical function related to the ability to capture good quality images of the mole to enable comparison over time.

### Technical Challenges of Using the Apps

In addition to such key aspects of image capture, the participants discussed other important technical challenges they experienced. A particular one, relevant to skin self-monitoring more broadly, is viewing moles in less accessible parts of the body, including the back. For many, this required seeking assistance from a partner or carer but was a greater challenge for those who lived alone.

Although all participants were regular users of smartphones, there were varying levels of reported proficiency in their use. Some were, therefore, not confident enough to use the app in the way it was intended. There were concerns related to this issue about the amount of time needed to learn how to use the individual apps and maintain the photos.

Participants also experienced specific technical issues with the apps; some participants complained about the apparent impact on battery life, whereas others had difficulties reinstalling the app when purchasing a new phone.

### App Reminders and Skin Self-monitoring Routines

All the apps had a reminder function to prompt users to examine their skin. There was mixed feedback on these reminders. Most participants thought they were helpful and used them to help keep on track with monitoring their skin. However, there were problems with the reminders not coinciding with individuals’ skin self-monitoring routines. For younger participants who were less regular with conducting skin self-monitoring, the app reminders were insufficient to prompt them to check their skin.

### The Apps and Their Positioning Within Existing Relationships With HCPs

Participants discussed the importance of the HCPs involved in managing their skin, and this often involved seeing multiple doctors, even for those participants recruited in primary care who had not been previously diagnosed with melanoma. Many participants spoke to their GPs regarding concerns about a specific mole, and some participants also attended primary care skin clinics; those with a previous melanoma also consulted their specialists for signs of recurrence and a whole-body examination. Participants also discussed how the app fit into these relationships with their HCP and how they could share and discuss the photographs they had been taking.

They felt that the ability to compare photographs over time within the app and have all their photographs stored in a single accessible place could help communicate with their doctors.

However, some participants felt that there was no place for the app because they were already being monitored closely by their doctors.

Related to this was the issue of greater trust in continuing to see their doctor than relying on an app. This model of care provided them with greater peace of mind and was more effective for the early detection of melanoma.

Others thought the apps were potentially more relevant to a rural audience, who did not have such good access to health care.

There was some support for the potential use of the apps to enable a telehealth model and change the way they interacted with their health professionals about their skin. They supported the idea of sending images directly to a specialist through an app for review, whereas others were more skeptical about this model of care. By assuming that even if they did send a photograph in for review, they would be asked to consult a doctor every time.

## Discussion

### Principal Findings

To our knowledge, this is one of the first studies to assess the experiences of people at higher risk of melanoma using mobile apps for skin self-monitoring. This qualitative study found that participants were receptive to the potential benefits of using mobile apps for skin self-monitoring. Not all participants engaged on a monthly basis with the use of apps, despite acknowledging their potential benefits. This is related to technical and practical barriers, including infrequent use limiting learning about app use. Additional barriers to adoption were the relationship of the apps to existing skin self-monitoring routines and skin checks provided by HCPs.

We found that perceptions of the quality of the apps were integral to its use and how it was experienced. Technology literacy was highly variable; although almost all participants used their smartphones regularly, they did not necessarily perceive the apps to be easy to use. Although some of these technical barriers could potentially be overcome by better app design, we must recognize the practical challenges related to the specific task; obtaining a high-quality image of a skin lesion, especially in certain parts of the body, is difficult, more so without assistance.

To our knowledge, this is the first qualitative study reporting the lack of actual use of skin self-monitoring apps in people at increased risk of melanoma. Only a quarter of the participants regularly used the app for the entire duration of the study. This was in an at-risk population who already had an increased personal incentive to use these melanoma skin self-monitoring apps. It is possible that even fewer people would actively engage with mobile app use among people at population-level risk. A recent randomized controlled trial of skin self-monitoring app use among a UK primary care population who were at increased risk of melanoma found no evidence of increased consulting about skin lesions over a 12-month period [21]. This study was unable to collect data on the actual use of the mobile app; however, according to our findings, the lack of effect in that trial may well have been due to limited engagement with the app.

Previous qualitative research has focused on patients’ intentions and attitudes toward using skin self-monitoring apps [14,22]. Dieng et al [22] interviewed patients who had a previous diagnosis of melanoma and asked about the possible use of digital technology to assess changes in skin lesions over time. Similar to our findings, participants had positive attitudes toward this type of technology and thought it would prompt them to visit their HCP if a concern was found ahead of their regular appointments. Our study suggests a large gap between intentions and actual engagement with the currently available skin self-monitoring apps.

Our study has highlighted the many technical and practical factors at play when patients experience skin self-monitoring apps. It emphasizes the importance of participants’ personal circumstances and their context as to whether they engage with these apps. It is important to understand patients’ existing relationships with HCPs and their access to regular clinical skin examinations, their current skin self-monitoring routines, and the role of partners or carers for assistance using the app. Only a minority of people in our study were regular users of these apps after 3 months. We do not know if they continued to use them for longer-term skin self-monitoring, but we suggest that both personal and contextual issues as well as the app-related technical issues are likely to determine this. This is echoed in many studies on health apps more broadly, where uptake is low and dropout is high. This has been observed in mental health apps [23], asthma apps [24], and diabetes apps [25]. Using depression health apps as an example, the completion of apps within the real-world setting was as low as 1%-28% [23].

### Strengths and Limitations

We conducted qualitative interviews in a relatively large sample, providing a rich, in-depth understanding of the factors influencing app use.

We recruited participants from two different populations: those at increased risk of melanoma in the general practice setting and those who have had a previous diagnosis of melanoma in the hospital setting. Both populations represent potential target users of these apps. We had initially expected app engagement to be higher in those with a previous melanoma but found that this may not hold true.

There were some limitations to this study. Although we recruited a large sample for a qualitative study, we experienced moderate attrition. A third of the participants withdrew or were lost to follow-up before completion, likely representing people who were even less inclined to engage with the apps. Nonetheless, it is clear that the sample we interviewed did not represent a self-selected group that was highly motivated to use these apps.

Considering the use of commercially available apps, we were unable to record the exact amount of time or the frequency of actual interactions with the apps used during the study and relied on self-reporting. We had no control over changes to app functions or updates. Therefore, we deliberately monitored use for a relatively short period of follow-up, which limits our understanding of or additional barriers to long-term adoption.

Finally, the participants themselves did not choose the apps but were only given 2 apps to try on the basis of their phone’s operating system. We do not know how the public currently selects skin self-monitoring apps from app stores or how payment for an app might influence whether users persevere with them for longer.

### Conclusions

This qualitative study provides important new findings about engagement with skin self-monitoring apps in people at increased risk of melanoma. The findings can make useful contributions to designing future apps or interventions for promoting skin self-monitoring. If such apps are to play a role in the early detection of melanoma, we must move beyond a focus on app design and diagnostic accuracy. This will require acknowledgment of the complex contextual factors affecting app use and incorporating app-based skin self-monitoring into existing models of care and skin assessments.

## Appendix

Multimedia Appendix 1 Interview guide.

Multimedia Appendix 2 Participant quotes table.

## Abbreviations

GP: general practitioner
HCP: health care provider
REDCap: Research Electronic Data Capture
