# Supplementary material for: Patients’ Experiences of Using Skin Self-monitoring Apps With People at Higher Risk of Melanoma: Qualitative Study
Source: JMIR Dermatol. 2021 Aug 13;4(2):e22583. doi: 10.2196/22583 (PMC10334956; doi:10.2196/22583)
Supplement: Multimedia Appendix 1 [file derma_v4i2e22583_app1.docx]

Appendix 1: Interview guide

Melanoma Apps Study: Skin self-monitoring app assessment by people at increased risk of melanoma

INTERVIEW QUESTIONS:

1. What are the apps you commonly use and are any of them health related?
2. Tell me a bit about your experiences of using the apps on your phone. Do you have a preference for a particular app?
3. What aspects of each app do you like? Why?
4. Are there certain aspects of the apps that you didn’t like? Why?
5. Did you use both apps the whole three months? If so, why (not)?
6. Did you visit a health professional about a newly concerning mole during the last three months? If so, what happened? Was it a mole you were monitoring with the apps?
7. Will you continue to use both or one of the apps after this study? If so, why?
8. Did you monitor your own skin outside of the use of the apps? If so, how?
9. [Secondary care participants only] How do you think previously having a melanoma has changed your behaviour towards your skin health?
10. [Secondary care participants only] How did was your previous melanoma discovered and diagnosed?
11. [Apart from your personal experience of melanoma] have any family members or close friends been affected by skin cancer or melanoma?
