# Supplementary material for: Patients’ Experiences of Using Skin Self-monitoring Apps With People at Higher Risk of Melanoma: Qualitative Study
Source: JMIR Dermatol. 2021 Aug 13;4(2):e22583. doi: 10.2196/22583 (PMC10334956; doi:10.2196/22583)
Supplement: Multimedia Appendix 2 [file derma_v4i2e22583_app2.docx]

Multimedia Appendix 2. Participant quotes

| **Theme** | **Participant quote** |
| --- | --- |
| **Benefits of early detection** | “*can help and improve cancer patient’s behaviour to detect their melanomas*” [Male, aged 57 years, secondary care]  “*if we use the app regularly, every two or three months, take a photo and compare it to a previous one and hopefully that might detect something earlier*.” [Male, aged 58 years, secondary care]  “*it’s sort of a little bit of peace of mind, on any that you’re a bit unsure of.*” [Female, aged 78 years, primary care] |
| **Experience of SSM** | “*I keep an eye on them here and there just to make sure they haven’t changed, sometimes some of them creep up on me but I just do the standard sort of check after a shower and in the mirror or something like that*” [Male, aged 70 years, primary care]  “*It’s more probably opportunistic when I’m, you know I’m drying myself off or you know I’m, you know I’m, about to go for a swim and I’m look and I’m like oh yeah that one*” [Male, aged 45 years, secondary care] |
| **Experience of using apps to support SSM** | “*Yeah it just looked like it was more user friendly and simpler to use and wasn’t too much detail, so, easier for people to remember how to use it the better, especially since we were only using it once in a while*.” [Female, aged 58 years, secondary care]  “*I found everything very intuitive. I didn’t have to think about it, it was just like yeah this is it. It was actually very, it is still very easy, I actually really liked using it because with these things you don’t want to think too much, especially to just take a picture. Look, especially my skin pal.*” [Male, aged 51 years, primary care]  “*It was just harder to, that one was harder to follow, you know I just didn’t understand it as easily as the first one, that one I understood… I couldn’t work out how then it said go to the next step and it didn’t go to the next step, and then sometimes it worked. Then I couldn’t work it out, it just sort of didn’t flow on.*” [Female, aged 58 years, secondary care]  “*The pictures were probably not up to the detail that you probably wanted to see the difference, if it had change or if it had you know a bit bigger, it was just a bit hard to do it. I don’t know why but the camera worked a lot better on one app and not the other. I just more or less gave up with that app because I was getting really frustrated*.” [Male, aged 45 years, secondary care]  “*Yeah because you had to move the phone in and out to get it into the right spot for it to be able to pick up whatever it was the correct spot for the photo I think, and it just really wouldn’t work.*” [Male, aged 45 years, secondary care] |
| **Technical challenges of using the apps** | “*It’s difficult, especially for people that don’t have someone else to, you know, take pictures of the back of their leg and back of back.”* [Female, aged 58 years, secondary care]  “*Yeah because most of my bad moles are on the back so and I don’t have a stick. To take pictures of my back it is beyond my ability to take pictures on my back. So, I suppose what makes it a bit awkward, but you really do need two people to do this*.” [Male, aged 42 years, primary care]  “*I’m not computer literate and I’m not, to be honest I’m not in the slightest bit interested in the computer. I will send an email; I’ll send a message and I take photos and that’s about it… If you are not used to using them all the time it can be a challenge*.” [Female, aged 73 years, primary care]  “*I think I spent a couple of hours to understand the UM skin. I think it would be a bit demanding to expect someone to keep taking photographs*.” [Male, aged 77 years, primary care]  “*My Skin Pal did work at the start but when we changed phones, they downloaded everything for us but that one didn’t download and won’t work, and we don’t know why*.” [Male, aged 77 years, secondary care] |
| **App reminders and SSM routines** | “*Yeah, I was in bed and the thing has gone off and I’ve looked at it and gone oh, I need to do that mole thing and then it just never happened, sort of thing… I suggest if you had a time like 6pm or something when you get a reminder then, you might make, you might have more success of getting it done right then and there*” [Male, aged 48 years, secondary care]  “*It’s just like being too busy to go to the doctor that’s probably the thing that stops most people from using these apps a little bit more*” [Female, aged 31 years, secondary care]  “*It’s just one of those things you know you don’t really do or think about very often… and life sometimes gets in the way I guess.”* [Male, aged 32 years, primary care] |
| **The apps and their positioning within existing relationships with healthcare providers** | “*I’m meant to go every 6 months for a check-up at [hospital] and every three months for a check-up at my GP and every month I’m meant to do my own self check*” [Female, aged 31 years, secondary care]  “*If I saw something and used the app more frequently, then when I got to the health professional, I could be like, oh hang on I’ve got this app, this is what I took, and this is when I took it*” [Female, aged 61 years, secondary care]  “*it’s just, it’s handier when you’re got an app or something you can compare it to yourself and then if you’ve got it there you can actually take the evidence in with you.”* [Male, aged 42 years, primary care]  “*you’re getting checked anyway so it’s probably just a bit more pointed towards people that haven’t got it and could self-check that aren’t getting themselves checked*.” [Male, aged 45 years, secondary care]  “*Oh, like I said, I am pretty paranoid these days, it’s always in the back of your head… I want to rely on an app, but I just think I’d rather for peace of mind, I’d rather just go straight to the GP*.” [Female, ages 38 years, primary care]  “*unless you are maybe living out somewhere rural where there isn’t a doctor around possibly, but when we got doctors so close at hand it’s not so important*” [Male, aged 32 years, primary care]  “*I think it would ease a lot of peoples mind, you know, send through to have a look at this, and you could get a response within the day, that day or the next morning. I think that would, you know, help a lot of people and I would be happy to do that*.” [Male, aged 45 years, secondary care]  “*It sounds good, but I imagine that they’ll probably go, oh we would probably like to have a better look at it, and I would end up going in anyway.”* [Male, aged 32 years, primary care] |
